# Determination, distribution, and environmental fate of *Bacillus thuringiensis* spores in various honeybee matrices after field application as plant protection product

**DOI:** 10.1007/s11356-022-19414-5

**Published:** 2022-02-26

**Authors:** Abdulrahim T. Alkassab, Hannes Beims, Martina Janke, Jens Pistorius

**Affiliations:** 1 Julius Kühn-Institut (JKI), Federal Research Centre for Cultivated Plants, Institute for Bee Protection, Braunschweig, Germany; 2 Lower Saxony State Office for Consumer Protection and Food Safety (LAVES), Institute for Apiculture, Celle, Germany

**Keywords:** *Apis mellifera*, Microbial plant protection products, Non-target organisms, Microbial insecticide

## Abstract

The increasing use of *Bacillus thuringiensis* (Bt)–based plant protection products (PPPs) has recently raised some concerns regarding their environmental accumulation and possible chronic exposure of non-target species, including pollinators, to higher than expected doses. The exposure level of such microbial PPPs in bee’s matrices under field conditions has not yet been described. Therefore, the current study aims at evaluating the realistic exposure level and comparing the distributions and persistence of Bt spores under field conditions. A field trial with spray application in oilseed rape (*Brassica napus*) as a representative bee-attractive crop was conducted. During the experimental period, different matrices, including honeybee-collected and -stored matrices as well as bee larvae and dead bees, were collected and analyzed using newly established methods. The concentration of Bt spores in the various matrices was quantified. The results show high levels of Bt spores in honey sac and pollen pellets with reduction over time but no reduction of Bt spores in the stored matrices within the colony, i.e., nectar and bee bread, over time. Our results show for the first time the exposure level of bees to Bt spores under realistic field conditions and are fundamentally important for assessing potential exposure and risks for pollinators.

## Introduction

Nowadays, the growing world population requires constant high crop yields. To avoid losses caused by weeds, pests, and diseases, which can reach 37% of all potential crops (Pimentel 1997), farmers follow several approaches. Integrated pest management (IPM) is a general approach, including chemical plant protection products (PPPs) to suppress pest populations below the economic threshold (Peshin et al. 2009). However, increasing numbers of studies have shown the potential adverse effects of chemical PPPs on insect pollinators, including *Apis* and non-*Apis* bees, leading to a wide range of microbial pest-controlling products (MPCPs) being developed as more specific and safer alternatives (Köhl et al. 2019). Among the MPCPs, several products containing different strains and isolates of *Bacillus thuringiensis* (Bt) are applied worldwide as entomopathogen biocontrol agents against insect pests in agriculture and forest (Sanchis and Bourguet 2008; Lacey et al. 2015).

Currently, the commercial formulations based on different isolates of Bt subsp. *kurstaki* (Btk) and Bt subsp. *aizawai* (Bta) to control lepidopteran larvae are the most sprayed bioinsecticides in organic and conventional farming (Bravo et al. 2011). These products are recommended to be sprayed repeatedly within a short interval (3–8 days) due to the sensitivity of toxin crystals or spores to abiotic conditions like UV (EFSA Biohaz Panel (EFSA Panel on Biological Hazards), 2016). The formulations contain different compounds to protect spores and toxin crystals (Brar et al. 2006). Previous studies reported that Btk can persist on the leaves’ surface over 72 h after application, decreasing within 28 days to the environmental background level (Bizzarri and Bishop, 2008; Raymond et al. 2010).

The increasing use of Bt–based products has recently raised concerns regarding environmental Bt accumulation which can lead to chronic exposure of non-target species, including entomophagous insects and pollinators, to higher doses than expected (Babin et al., 2020). Bt is known for synthesizing a wide range of toxins encoded on large plasmids. Therefore, each subspecies and/or strain can harbor different plasmids, encoding for synthesized toxins related to their biological activity and the potential target insects (Palma et al. 2014). The most studied insecticidal toxins are Cry-toxins as δ-endotoxins affecting the susceptible insects after oral uptake (Bravo et al. 2011 and Bravo et al., 2017; Mendoza-Almanza et al. 2020). Several reports regarding direct and indirect cross-effects of Bt formulations and their toxins across insect taxa and orders recently indicated the semi-specificity of Bt (van Frankenhuyzen 2017; Redmond et al. 2020; Coyle et al. 2000; Babin et al. 2020; Nawrot-Esposito et al. 2020; Tudoran et al. 2020). Lepidopteran-targeted formulation, containing Bta ABTS 1857, has demonstrated increasing mortality of adult and larvae of honeybees after chronic exposure under laboratory conditions (Steinigeweg et al. 2021).

Their foraging activity may expose bees to Bt products, collecting nectar and pollen contaminated after spray application during flowering on various crops and transporting them to the colony. In-hive conditions differ highly from field conditions, e.g., no more UV effects on the spores and/or the products along with higher humidity. With no information about the realistic exposure yet available, the viability and environmental fate of Bt spores in the collected matrices, i.e., nectar and pollen, after application in different bee-attractive crops have to be investigated. Recently, we considered the distribution of Bt spores within the colony in several matrices after an artificial in-hive feeding experiment, resulting in the presence of Bt in all matrices at different concentrations over 2–3 weeks (Steinigeweg et al., 2021).

The current study aims at (1) evaluating the realistic exposure level under field conditions after spray application in oilseed rape as a representative bee-attractive crop and (2) comparing the distributions and persistence of Bt spores in different bee-collected and -stored matrices. These results will be of great importance to assess potential exposure and risks for pollinators.

## Materials and methods

### Experimental setup and sample collection

The study was conducted in Celle, Germany, in 2020. Paired fields with treated and untreated winter oilseed rape (*Brassica napus*) were used as representative bee-attractive crops. The distance between both fields was approximately 2.5 km.

**Fig. 1:**
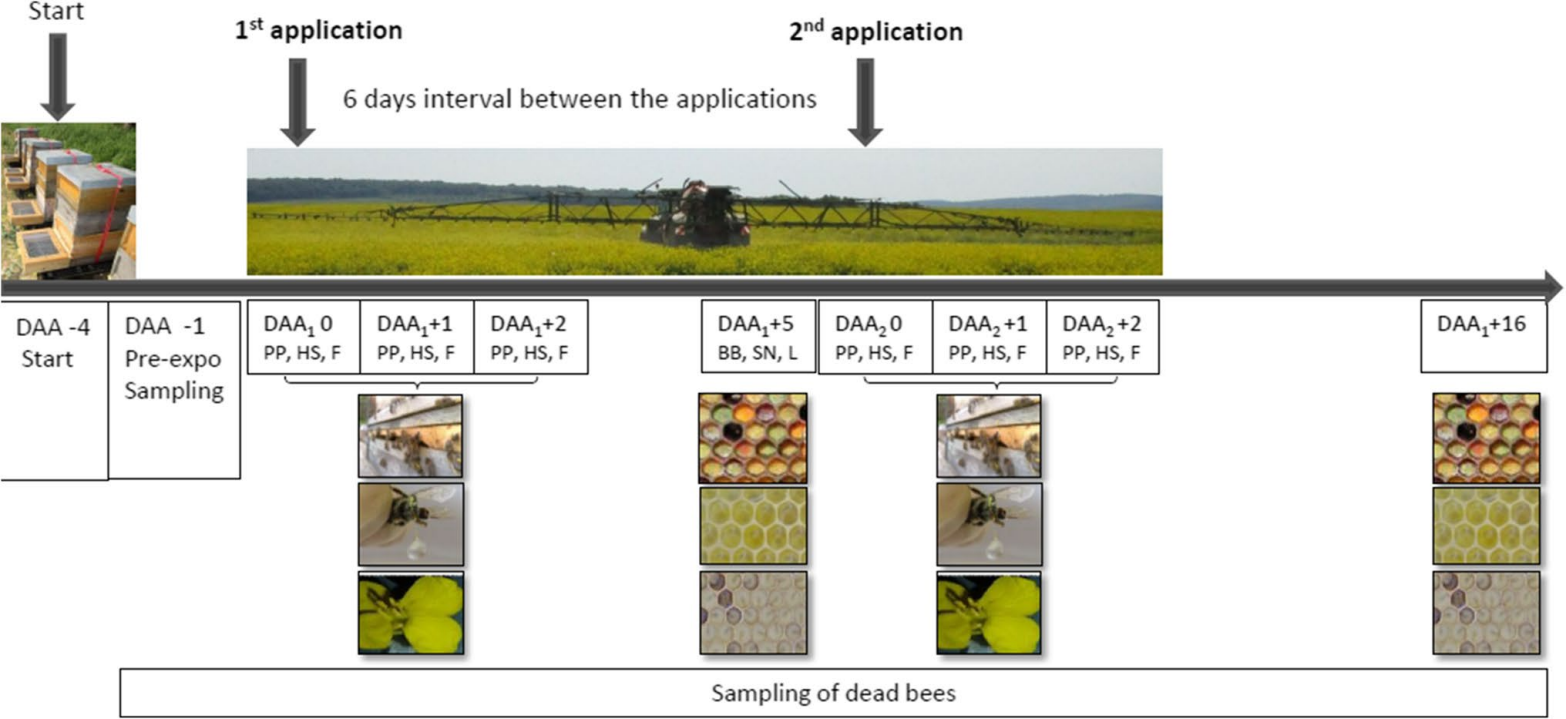
Experimental design and sampling dates. Seven experimental honeybee colonies were set up as a block on the edge of each oilseed rape field 4 days before the oilseed rape was in full bloom. The Bt–based product containing Bta strain ABTS 1857 was applied during full flowering twice with an interval of 6 days. At the end of flowering, i.e., 16 days after exposure (day after application (DAA), DAA_1_ + 16), the colonies were transferred off the field. Various matrices were collected, i.e., honey sac (HS), pollen pellets (PP), flowers (F), bee bread (BB), stored nectar (SN), and larvae (L)

Seven experimental honeybee colonies were set up as a block on the edge of each oilseed rape field. Figure 1 shows the time points of the field phase related to the application, i.e., day after application (DAA). The colonies were placed on the edge of the field, 4 days before the oilseed rape was in full bloom (DAA_1_ − 4; Fig. 1).

At full flowering of winter oilseed rape (BBCH 65), two applications (A_1_ and A_2_) were conducted with an interval of 6 days of PPP containing Bta strain ABTS 1857, simulating multiple recommended applications (Fig. 1). The maximum recommended rate of 1.5 kg/ha was applied using calibrated commercial sprayers delivering 900 L/ha, during bee flight. At the end of flowering (BBCH 68–69), i.e., 16 days after exposure (DAA_1_ + 16), the colonies were transferred off the field (Fig. 1).

Various matrices, including honey sacs and pollen pellets from forager bees, dead bees, larvae, bee bread, and stored nectar, were collected from each colony before application and at different times afterwards (Fig. 1). All samples were stored in the laboratory at − 20 °C until analysis. Approximately 50 foragers per sampling date were collected and then pollen pellets in the corbiculae were sampled. The honey stomach, i.e., honey sac, of each bee was sampled after dissection of the abdomen and separated from the rest of the digestive tract. The flowers were collected from 10 places from each field and pooled into one sample for each sampling time (Fig. 1). Furthermore, the dead bees were collected from dead traps (modified Gary trap (Gary 1960)) and pooled for each sampling time, pooling at least two bees from each colony to get a sample of approximately 1 g.

### Determination of Bt in the collected samples

Samples of different matrices were homogenized and dissolved 1:2 (*w/v*) in ddH_2_O. Tissue lysis was performed in Bead Tubes Type G (Macherey–Nagel, Germany) in a SpeedMill PLUS (Analytik Jena, Germany) for 30 s. Homogenates were used for serial dilution 10^−1^–10^−6^ in ddH_2_O in a final volume of 200 µL on an epMotion 5075 (Eppendorf, Germany). Triplicates of 75 µL per dilution were plated on LB agar (10 g trypton (Carl Roth, Germany), 5 g yeast extract (Carl Roth, Germany), 10 g NaCl (Carl Roth, Germany), 15 g agar (Carl Roth, Germany), and 1 L ddH_2_O) and incubated over night at 37 °C (Memmert HPP 750, Germany). Bt–typical colonies were finally quantified on each plate and their mean calculated (cfu/g).

### Identification of potential Bt colonies

Colonies were identified as Bt by qPCR, amplifying a partial sequence of the CryIAa gene (Ogunjimi et al. 2000; Steinigeweg et al. 2021). Accordingly, a random colony was resuspended in 50-µL ddH_2_O, followed by bacterial lysis at 95 °C for 15 min. Cell debris were sedimented at 5000 × *g* for 5 min, and 1 µL of supernatant was used as template for qPCR. PCRs were performed using the LUNA® Universal qPCR Master Mix (New England Biolabs, USA) according to the manufacturer protocol on an AriaMX Real-Time PCR system (Agilent, USA). Samples were identified as Bt–positive when Cq < 30 and 80 °C < Tm < 81 °C was obtained (Ruiz-Villalba et al. 2017).

### Statistical analysis

To analyze the differences between matrices over time, linear mixed models (LMMs) were used. Concentrations of Bt spores were set as dependent variables against sampling date and matrices as independent variables; the colony ID served as a random factor. All statistical analyses were conducted using software “R” version 4.0.3 “Bunny-Wunnies Freak Out” (R Core Team 2020) at the significance level of 0.05. Models were performed with the function lme from the nlme package version 3.1–152 (Pinheiro et al. 2021). Plots used the library ggplot2 (Wickham et al. 2020).

## Results and discussion

### Bt concentrations in fresh bee-collected matrices (nectar and pollen)

**Fig. 2:**
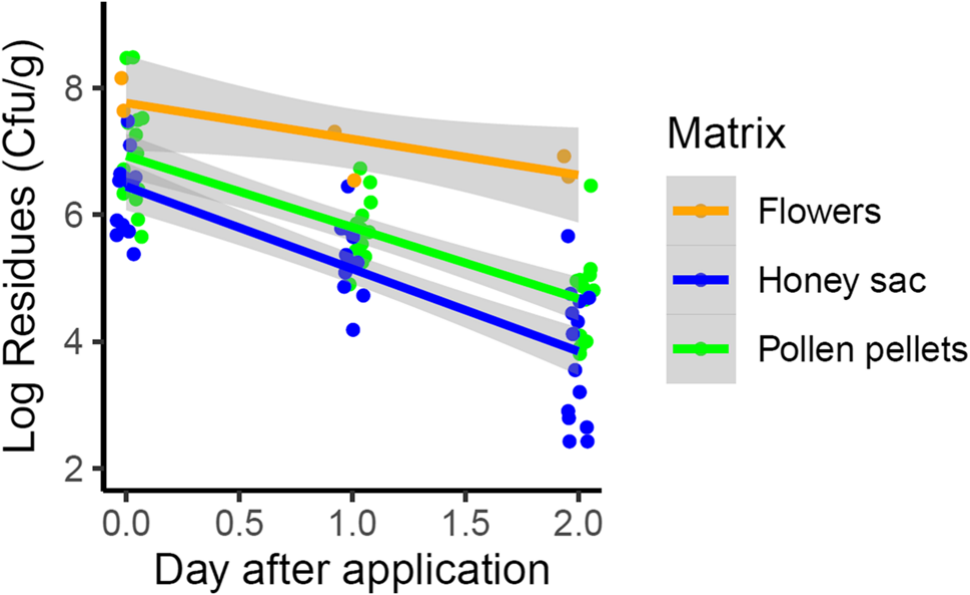
Concentrations of Bt spores as colony-forming units per gram matrix (cfu/g) over 2 days after application. Sample size is *n* = 7 colonies for honey sac and pollen pellets from about 50 foragers per sampling date. One pooled sample of flowers per date was analyzed. A significant decreasing concentration in all matrices over 2 days after applications was found (GLMM, *p* < 0.001). The level of Bt spores in the honey sac was significantly lower than in pollen pellets (GLMM, *p* = 0.045)

Our results show significantly decreasing concentrations in all matrices over 2 days after applications (Fig. 2; GLMM, *p* < 0.001). The level of Bt spores in the honey sac was significantly lower than in pollen pellets (Fig. 2; GLMM, *p* = 0.045). The maximum detected concentration in pollen pellets was 30.33 × 10^7^ cfu/g on DAA_2_ + 1 and 3.01 × 10^7^ cfu/g on DAA_1_ + 1 in the honey sac. Furthermore, the maximum detected concentration on the flowers of oilseed rape was 14.3 × 10^7^ cfu/g on DAA_1_ + 0 and 4.36 × 10^7^ cfu/g on DAA_2_ + 0 (Fig. 2). Overall, no significant differences were found between A_1_ and A_2_ regarding the detected concentrations (GLMM, *p* > 0.05). The concentrations spread among 10^5^–10^8^ cfu/g on the day of application and between 10^3^ and 10^5^ cfu/g after 2 days. No Bt spores were detected in samples collected before application from both fields or from the untreated field at subsequent dates, except in one sample of honey sacs with a trace concentration of 355 cfu/g indicating general low background concentrations of Bt.

Generally, few studies show persistence of Bt spores after application of Bt–based MPCPs in the environment, although wide use of Bt–based PPPs is approved worldwide. These PPPs are considered to have a low persistence on foliage under field conditions where the half-life period of viable Bt spores was assumed as a few hours and up to 2 days (Pinnock et al. 1971; Ignoffo and Garcia 1978; Pedersen et al. 1995; Haddad et al. 2005). The rapid degradation of Bt spores is reportedly related to several abiotic factors like UV radiation, temperature, and humidity (Dunkle and Shasha 1988; Ignoffo et al., 1974; Khorramvatan et al. 2014, Sansinenea et al. 2015); therefore, several attempts to develop formulations with better Bt stability under field conditions like UV-protective adjuvants sought to increase efficacy (e.g., Maghsoudi and Jalali 2017). The detected concentrations in our samples were still abundant at a high concentration > 10^6^ cfu/g 2 days after application though significantly decreased in the detected concentrations, confirmed by the results in bee-collected matrices (i.e., pollen and nectar), showing a significant decrease of the Bt concentration 2 days after applications.

### Bt concentrations in bee larvae and stored matrices (stored nectar and bee bread)

**Fig. 3:**
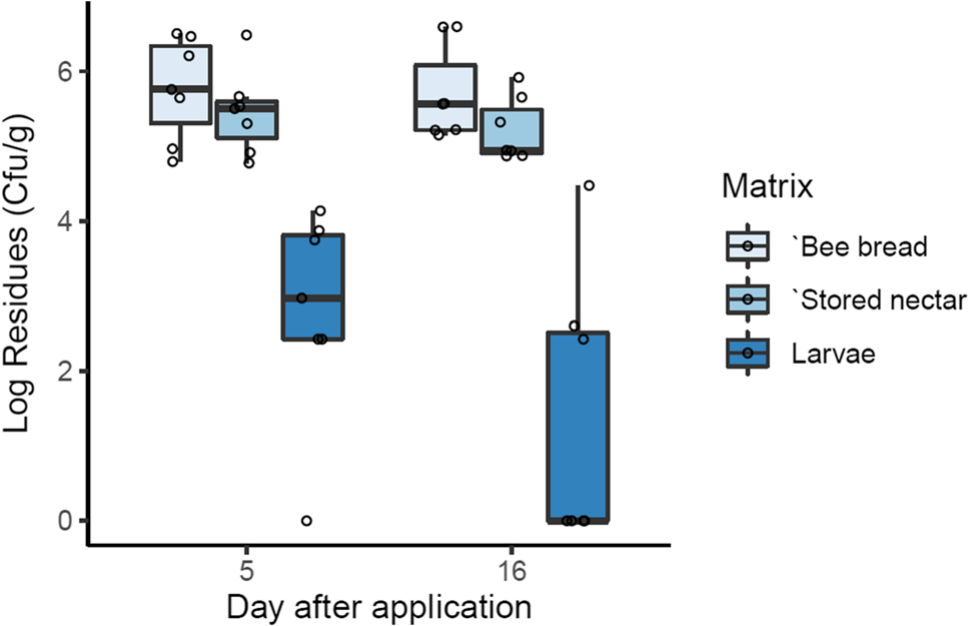
Concentrations of Bt spores in the stored matrices (nectar and pollen pellets) and bee larvae as colony-forming units per gram matrix (cfu/g) 5 and 16 days after first application. Sample size is *n* = 7 colonies per sampling date. No reduction of the Bt spores in the stored matrices, i.e., nectar and bee bread, within the colony over the sampling time was observed (GLMM, *p* > 0.05). The detected concentrations in bee larvae differed significantly from the stored matrices (GLMM, *p* = 0.009)

The results showed no reduction of the Bt spores in the stored matrices, i.e., nectar and bee bread, within the colony over the sampling time (Fig. 3; GLMM, *p* > 0.05). The maximum detected concentration in bee bread was 3.96 × 10^6^ cfu/g, whereas stored nectar presented a range (3.67 × 10^6^ cfu/g). These results indicate approximately 10 × lower concentrations in the stored than the freshly collected matrices. The detected concentrations in bee larvae spread in a smaller interval, i.e., from undetectable to 2.99 × 10^4^ cfu/g. Moreover, they differed significantly from the stored matrices (Fig. 3; GLMM, *p* = 0.009). No Bt spores were detected in all samples as well as in most samples from the colonies in the untreated field, except in two samples from one colony with trace concentrations 267 and 800 cfu/g.

These results concur with our previously published results (Steinigeweg et al., 2021). We found that the detected concentrations in stored food within the colony after feeding Bt–contaminated sugar solution ranged between 10^5^ and 10^6^ cfu/g. Nevertheless, we observed decreasing detected concentrations over the 20-day experimental period, perhaps related to the experimental procedure under semi-field conditions, giving the colony only 2 L of Bt–contaminated sugar solution and uptake to store this amount within a short period. Vandenberg and Shimanuki (1990) investigated the presence of viable spores following storage of the Bt–treated combs against wax moth (*Galleria mellonella* and *Achroia grisella*), reporting a long-term presence at ranges of 10^7^ cfu and 10^4^ cfu in comb pieces (5 × 10 cm) at 10 °C and 30 °C over 12 months storage, respectively. However, honey produced by bees on treated combs contained very low levels of viable Bt spores after 20 weeks. Thus, a long-term presence of Bt spores can be expected despite the low detected levels.

European Food Safety Agencies (EFSA) suggested the threshold of 10^5^ cfu/g on plant commodity at harvest time to cover the risk of food-borne poisonings (EFSA Biohaz Panel (EFSA Panel on Biological Hazards), 2016). Our results show the detected concentrations in collected pollen approximately 10^2^–10^3^ above this threshold. Moreover, the detected concentrations in stored nectar or later honey are mostly tenfold higher than the threshold, although 10 days after the second application.

The detected concentrations in larvae were significantly lower than the stored matrices, covering a range of 0–2.99 × 10^4^ cfu/g. Assuming that the L4 larvae weigh about 55–88 mg (Zółtowska et al. 2011), about 12–18 larvae represent the analyzed amount of 1 g; the maximum detected level of Bt spores can be assumed as about 10^3^ cfu/larvae. This level may still be tolerable for larvae, since they were alive during sampling. Steinigeweg et al. (2021) reported high larval mortality under laboratory conditions after exposure to a range of 0.16–32.00 µg product/larvae corresponding to concentrations of about 10^4^–10^6^ cfu/larvae. These concentrations are calculated based on the reported maximum concentrations of 6 × 10^13^ cfu/kg of Bta ABTS 1857 in the formulated product (EFSA, 2020). Therefore, further research is needed to assess the tolerable level of Bt for larvae.

### Bt concentrations in dead bees

**Fig. 4:**
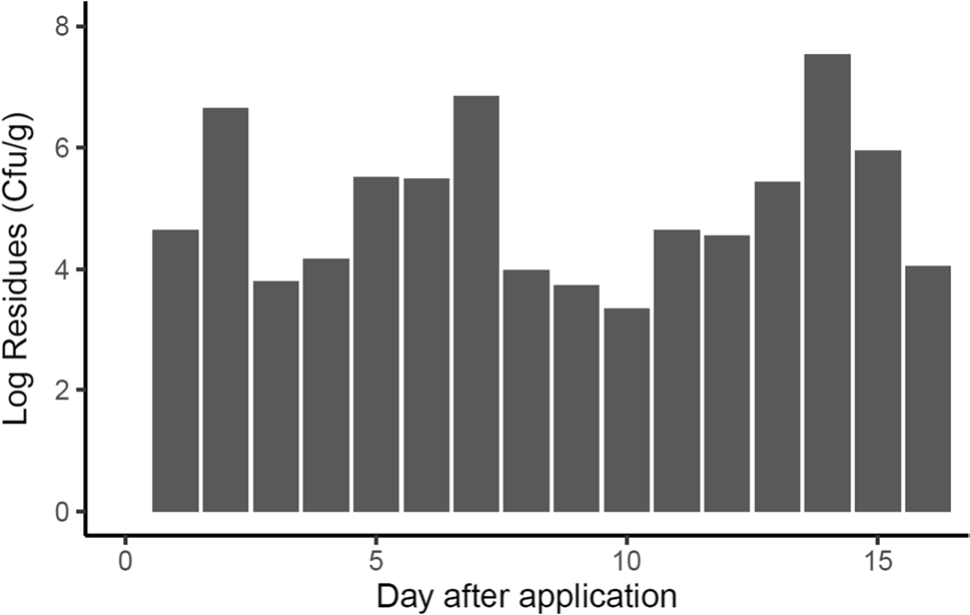
Concentrations of Bt spores in dead bees as colony-forming units per gram matrix (cfu/g) over the experimental period. Pooled sample from all colonies with about 10–14 bees per sampling date was analyzed. High concentrations of Bt spores were detected at a later date after applications, indicating the chronic Bt spore exposure of bees and possible infective properties of Bt

Bt spores were detected in all samples collected from the treated field after applications. The concentrations ranged from 2.23 × 10^3^ cfu/g to 3.42 × 10^7^ cfu/g (Fig. 4). Remarkably, high concentrations of Bt spores were detected at a later date after applications, indicating the chronic Bt spore exposure of bees and possible infective properties of Bt. No Bt spores were detected in all samples taken from the treated field before application or from the control field at all sampling dates.

Although Bt strains are assumed to be selective on target pests, recent reports indicate cross-order effects (van Frankenhuyzen et al. 2017). For example, the Coleopteran-targeted formulation can cause negative effects on Lepidopteran (Redmond et al. 2020), the Lepidopteran-targeted formulation on Coleopteran and the Dipteran (Coyle et al. 2000; Babin et al. 2020; Nawrot-Esposito et al. 2020), and Diptera-targeted formulation on Coleopteran (Tudoran et al. 2020). Regarding the effects on bees, differences in the sensitivity of adult worker bees to different Bt products were reported (Brighenti et al. 2007; Mommaerts et al. 2010; D’urso V, Mazzeo G, Vaccalluzzo V, Sabella G, Bucchieri F, Viscuso R, Vitale DG, , 2017; Libardoni et al. 2018; Potrich et al. 2018; Steinigeweg et al. 2021). Steinigeweg et al. (2021) reported increased mortality under laboratory conditions after exposure to Bta ABTS 1857 at a range of 10^6^–10^7^ cfu/g. Other Bt strains also reduced the survival duration of adult bees (Libardoni et al. 2018; Potrich et al. 2018). Studies considered the interactions between single species in the bee gut microbiome and their community dynamics in relation to the bees’ health (Engel et al. 2016). Since the applied MPCPs contain living active ingredients, further studies should particularly determine their impact on the composition and development of the honeybee gut microbiome.

## Conclusion

Our study shows for the first time that the viability of Bt spores differs from honeybee colony conditions to field conditions. Remarkably, although the most used microorganism isolated from the natural environment, the application intensity and formulated products may cause significantly higher exposure levels than the background level. Thus, exhaustive examinations of MPCPs’ effect on the honeybee and other bee species under field conditions will help understand the natural role and the behavior of “living active ingredients” for beneficial organisms. This should include several parameters to be long-term–investigated to enable proper risk assessment.

## Data Availability

The datasets used and/or analyzed during the current study are available from the corresponding author on reasonable request.
